# Effects of in ovo taurine administration on cyclic heat stress in broiler chickens

**DOI:** 10.1016/j.psj.2026.106450

**Published:** 2026-01-14

**Authors:** Vaishali Gupta, Yun-Ji Hwang, Meg Aui Delmonte, Chris Major Ncho, Yang-Ho Choi

**Affiliations:** a Department of Animal Science, Gyeongsang National University, Jinju 52828, South Korea; b Division of Applied Life Sciences (BK21 FOUR Program), Gyeongsang National University, Jinju 52828, South Korea; c Institute of Agriculture and Life Sciences, Gyeongsang National University, Jinju 52828, South Korea

**Keywords:** In ovo feeding, Taurine, DNA-methylation, Antioxidants, Heat stress

## Abstract

High temperature is a known abiotic stressor in broiler chickens, causing oxidative damage and altering gene expression. The present study was conducted to study the role of in ovo feeding of taurine against heat-induced damage in the broiler chickens. It was hypothesized that pre-hatch supplementation with taurine induces epigenetic changes such as DNA methylation and demethylation, which could help develop resistance to heat stress (HS) at later stages of life. For this, at 17.5 days of incubation, 360 fertile eggs from 37-week-old Arbor Acre breeder hens were divided into four groups: injected with distilled water (0TAU) × 2, and others injected with taurine at 1 %, 3 %, or 5 % concentrations (1TAU, 3TAU, 5TAU). For the in ovo feeding, a 23-gauge needle was used to deposit 0.6mL of solution into the amniotic sac. During rearing days 29 to 34, broiler chickens were exposed to a cyclic heat stress (HS, 31 ± 1 °C, 8 hours) or kept at a thermoneutral temperature (TN) zone (21 ± 1 °C). Hence, the treatment groups were: (i) 0TAU-TN, (ii) 0TAU-HS, (iii) 1TAU-HS, (iv) 3TAU-HS, and (v) 5TAU-HS. While the organ indices, average daily feed intake (ADFI) and feed conversion ratio (FCR) did not differ significantly, in ovo taurine linearly increased average daily gain (ADG) during the heat stress (HS) period (p = 0.032). The 2,2-diphenyl-1-picrylhydrazyl radical scavenging activity% (DPPH-RSA%) in plasma showed a linear increase (p = 0.001) with taurine doses. Among the studied plasma metabolites, only alanine transaminase (ALT) was significantly affected, being lower in 1TAU-HS and 3TAU-HS compared to 5TAU-HS (p = 0.022). Individual gene expressions showed no significant variation across treatments. However, a planned contrast revealed upregulation of DNA methylation genes in the 5TAU-HS group compared to the 0TAU-TN group (p = 0.030). Strong positive correlations were observed among DNA methylation, demethylation, and NADPH oxidase (NOX) -related genes, suggesting coordinated regulation. Negative correlations between MDA and antioxidant enzymes indicated oxidative stress-related damage under HS. Hence, taurine linearly improved ADG under HS. While it did not significantly influence individual gene expression, 5TAU upregulated the overall DNA-methylation-related genes, suggesting a possible long-term adaptive response under HS.

## Introduction

Poultry products and meat have witnessed a surge in demand due to their quality and affordability. Hence, it significantly contributes to global food security (Sheffield et al., 2024). However, the expansion of the poultry industry is faced with several challenges, including climate-induced heat stress (HS), which is one of the major stressors. HS has become an even more pronounced menace in the tropical and subtropical regions (Lara and Rostagno, 2013). Broiler chickens are highly susceptible to HS because of their high metabolic rate (Yahav, 2009) and a limited ability to dissipate heat due to the absence of sweat glands (Gupta et al., 2024). Repeated and prolonged exposure to HS impairs growth (Goel et al., 2023), alters physiochemical properties, oxidative balance, and suppresses immunity, while also deteriorating meat quality (Mir et al., 2017). At the cellular level, the biochemical characteristics of the cell are disrupted due to the overproduction of reactive oxygen species (ROS) (Huang et al., 2015). Although ROS are generated daily during various metabolic activities, under HS, the pace of ROS generation exceeds the pace of their degradation (WM, 2010). Hence, causing oxidative imbalance and damaging the macromolecules such as lipids, proteins, and amino acids (Lin et al., 2006). The oxidative stress caused by these ROS accelerates inflammation, affecting cellular function, overall health, and performance of the birds (Goel et al., 2022).

Considering the role of taurine, a non-proteinogenic, sulfur-containing amino acid, in combating cellular stress (Uyanga et al., 2022), it has been widely studied for various biological functions. Under stress-free conditions, taurine is considered a non-essential amino acid synthesized in the body by utilizing methionine and cysteine (Ripps and Shen, 2012). It is a β-acid which forms a significant pool of free amino acids in cytosol (Fukuda et al., 1982). In addition to maintaining cellular homeostasis and osmoregulation, taurine also protects cells against oxidative stress and inflammation (Ripps and Shen, 2012). The cellular antioxidant defense machinery, including enzymes such as superoxide dismutase (SOD), catalase (CAT), and glutathione peroxidase (GPX), is strengthened via taurine supplementation (Han et al., 2020b; Huxtable, 1992). In the liver, taurine reduces lipid accumulation and improves circulating serum triglycerides (Chang et al., 2011; Gentile et al., 2011; Han et al., 2020b; Nardelli et al., 2011). Hence, owing to the antioxidant and cytoprotective properties, taurine (upto 5 %) could be a beneficial amino acid to supplement under HS in broiler chickens. Broiler chickens have responded positively to the supplementation of taurine in feed (Han et al., 2020a; Lee et al., 2004; Ma et al., 2021; Xu et al., 2020). Dietary supplementation of taurine improved lipid metabolism and growth in broiler chickens (Lee et al., 2004). Under lipopolysaccharide-induced oxidative stress, it protected the cells from oxidation and inflammation (Han et al., 2020a). Dietary supplementation of 5 % taurine was effective in improving carcass characteristics in broiler chickens (Lu et al., 2019a). In ovo supplementation of taurine has been shown to mitigate hatching stress (Baykalir et al., 2024). In the pectoral muscles of developing hen embryos, the concentration of carnosine and anserine was significantly increased following 50 – 500 ppm of in ovo taurine feeding (Łukasiewicz Mierzejewska et al., 2024). Further, nano particles of taurine injected at 3 days of incubation enhanced the number of cells in the breast muscle of broiler chicken embryos (Zielińska et al., 2010).

The hatchability and post-hatch growth and immunity of the chickens are affected by the optimal nutritional supply to the embryo (Vieira and Moran Jr, 1999). Several studies have shown that supplementation of extra nutrients during the late embryonic development (during days 17 – 18) was beneficial to enhance hatchling weight, immunity and metabolic efficiency in chickens (Araújo et al., 2019; Das et al., 2021; Fatemi et al., 2020; Lotfi et al., 2013; Meijer et al., 2025; Nabi et al., 2024; Uni et al., 2005; Zhu et al., 2019). Considering that taurine transporters are expressed during embryonic phases in mice and chickens (Kim et al., 2006), it suggests that taurine may be actively utilized by developing embryos. Hence, the supplementation of taurine during embryonic development in broiler chickens could be beneficial for physiological reprogramming the embryos.

The current study was designed to study the post-hatch effectiveness of in ovo taurine supplementation (up to 5 % at 17.5 embryonic days of development) to the broiler chickens under cyclic HS. The study was made comprehensive by including the study of growth alongside plasma biochemicals and antioxidant profile under HS conditions. Furthermore, hepatic expression of antioxidative, DNA-methylation and DNA-demethylation related enzymes was evaluated to understand the early life programming effect of taurine on broiler chicken embryos. This study examined whether in ovo taurine improves growth, metabolic and hepatic responses, and antioxidant status to enhance heat-stress resilience in broiler chickens.

## Materials and methods

The study was conducted in the animal research facility of Gyeongsang National University (GNU), Jinju-si, South Korea. The study protocol was approved by the Institutional Animal Care and Use Committee of GNU (GNU-250623-C0137).

### Incubation and in ovo feeding protocol

Fertilized eggs laid by 37—week—old Arbor Acre breeder hens were procured from a commercial hatchery (Harim Hatchery, Iksan, South Korea). All the eggs were numbered and weighed individually, and then incubated in a Rcom Maru CD 1000 deluxe incubator (Rcom Co., Ltd., Kimhae, Korea). The temperature and relative humidity were maintained at 37.8 °C and 56 % respectively, until 18 days of embryonic development (ED). Thereafter, a temperature of 36.8 °C and 70 % relative humidity were maintained in the hatcher until the day of hatch. Taurine was obtained from Sigma-Aldrich (#T8691-25G, Sigma-Aldrich, St. Louis, MO, USA) and dissolved in distilled water (DDW) to prepare 1 %, 3 %, and 5 % solutions directly. At ED17.5, 360 eggs (64.36 ± 4.22 g) were equally distributed into four groups. The first group consisted of 144 eggs injected with distilled water (DDW) or 0 % taurine (0TAU), while the remaining three groups (with 72 eggs each) were injected with 1 % (1TAU), 3 % (3TAU), and 5 % taurine (5TAU) solutions, respectively. During rearing, the 0TAU treatment was further divided into two groups to be raised at thermoneutral (TN) and cyclic heat stress (CHS) temperatures.

For in ovo feeding, a small section of the egg was disinfected with a swab of 70 % alcohol. Next, a tiny hole was created using a dental drill (Saeshin, Daegu, Korea). This was followed by the deposition of 0.6mL of solution into the amniotic sac of the embryos using a hypodermic syringe with a 23-gauge, 1-inch-long needle. A surgical tape (3M Micropore, Saint Paul, MN, USA) was used to seal the hole and prevent infection. The protocols have been detailed in our previous studies (Gupta et al., 2022; Ncho et al., 2021a).

### Rearing and heat stress protocol

The hatched chicks were visually inspected for activity and alertness. A total of thirty-six active chicks per treatment were randomly divided into six cages, each containing six birds. For the study, each cage represented a replicate. The cage distribution in the animal house followed a randomized block design. The chicks were raised under standard temperature and humidity guidelines (Arbor Acre pocket guide) until 4 weeks of age. Between the ages of 29 and 34 days, four birds were housed per cage. One group of 0TAU, 1TAU, 3TAU, and 5TAU were subjected to a CHS temperature of 31 ± 1 °C, while the other group of 0TAU was reared at a standard thermo-neutral temperature (TN) of 21 ± 1 °C, as previously described (Gupta et al., 2024). In the CHS room, the temperature was increased steadily to reach 31 ± 1 °C in 3 hours. This peak temperature was maintained for next 3 hours, followed by drop in the temperature in the next 30 minutes. Relative humidity in both rooms was maintained at 50 ± 5 %. Hence, the treatment groups in this study were: (i) 0TAU-TN (injected with DDW, reared at TN temperature); (ii) 0TAU-HS (injected with DDW, reared at CHS temperature); (iii) 1TAU-HS (injected with 1 % taurine, reared at CHS temperature); (iv) 3TAU-HS (injected with 3 % taurine, reared at CHS temperature); (v) 5TAU-HS (injected with 5 % taurine, reared at CHS temperature).

**Fig. 1:**
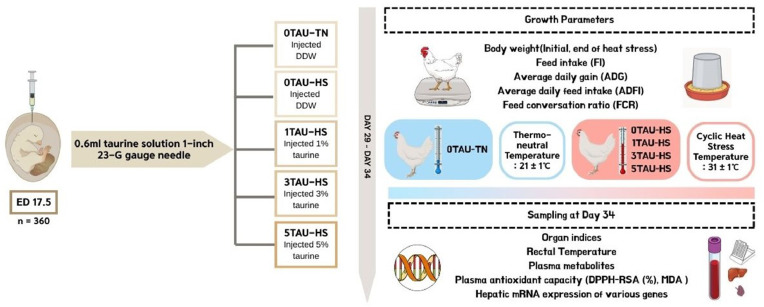
Diagrammatic representation of experimental design.

During rearing, birds were fed with a commercial ration for two phases (i) 0-3 weeks, and (ii) 4-5 weeks (until the end of the study). The feed was procured from Nonghyup Feed (Broiler Luxury, Nonghyup Feed, Gyeongju, South Korea). The feed composition has been detailed in supplementary Table 1. Feed and water were provided ad libitum. The birds were weighed before (BW initial) and at the end of heat stress (BW final), and feed intake (FI) was recorded. Average daily gain (ADG), average daily feed intake (ADFI) and feed conversion ratio (FCR) were calculated based on body weight (BW) and FI. The overall experimental protocol has been illustrated in Fig. 1.

### Collection of samples and rectal temperature

At the end of CHS at day 34, one bird per cage was randomly selected for biological sample collection. The euthanization of the birds was performed in a carbon dioxide chamber. Blood samples (5 mL) were collected in heparinized vacuum containers (#367874, BD Co., Ltd., Franklin Lakes, NJ, USA) and centrifuged at 2000 × g for 10 minutes to separate the plasma. Liver, spleen and Bursa of Fabricius were excised and their weights were recorded. The relative weight of organs was calculated as:

Relativeweight(%)=(Absoluteorganweight/Bodyweightofthebird)×100(%)

A small section of the liver was collected in tissue cassettes and snap-frozen in liquid nitrogen. Later, the frozen samples were transferred to a −80°C refrigerator and stored until processing.

One bird per cage was randomly selected, and a digital thermometer (HI 91610; Hanna Instruments Inc., Padova, Italy) was used to measure the rectal temperature (RT). The probe was inserted 3 cm deep into the rectum, and the temperature was recorded at the start and end of the HS period.

### Plasma antioxidant capacity and metabolites estimation

General metabolites such as glucose, triglycerides, cholesterol, liver enzymes such as alanine transaminase (ALT), aspartate aminotransferase (AST), and protein profile in terms of total protein, albumin, globulin and albumin: globulin ratio in plasma were measured using the Catalyst one chemistry analyzer (IDEXX Co., Ltd., Westbrook, ME, USA) following the manufacturer’s guide.

The plasma free radical scavenging activity was assessed using a 2,2-diphenyl-1-picrylhydrazyl radical scavenging activity assay (DPPH-RSA%), with a slightly modified protocol described previously (Gerasopoulos et al., 2015). Briefly, 20µL plasma was diluted with 480µL sodium-potassium buffer. An equal volume of 0.1mM DPPH reagent was added to the diluted mixture and incubated in the dark for 30 minutes. Thereafter, the solution was centrifuged at 10,000 × g for 6 minutes. Finally, the absorbance of the supernatant was read at 517 nm. The DPPH-RSA (%) was calculated as:

DPPH−RSA(%)=[(A1−−A0)/A0]×100%

Where A0 represents the absorbance of the blank sample (buffer and DPPH reagent without plasma), and A1 denotes the absorbance of the test sample.

In the plasma, the concentration of the lipid peroxidation product malondialdehyde (MDA) was measured using previously described methods (Jyothi et al., 2008). Briefly, 400µL plasma was mixed with an equal amount of 40 % trichloroacetic acid (TCA; # 76-03-9, Merck, Sigma-Aldrich, St. Louis, MO). Next, 0.67 % thiobarbituric acid (TBA; # 504-17-6, Merck, Sigma-Aldrich, St. Louis, MO) was added to an equal volume of the mixture. The mixture was vortexed well for uniform mixing. Thereafter, the tubes were kept in a water bath maintained at 95 °C for 45 minutes, followed by cooling in an ice bath for 5 minutes. After cooling, the sample tubes were centrifuged at 10,000 × g for 6 minutes, and the supernatant was used to measure the absorbance at 530 nm. The MDA content was estimated using the formula:

MDA(μmol/L)=A/(K×L)

Where A is the absorbance measured, K denotes the molar extinction coefficient (1.5 × 10^5^), and L is the length of the cuvette path (1 cm).

The antioxidant balance was calculated as the ratio of DPPH-RSA (%) to MDA content, based on our previous studies (Gupta et al., 2024).

### Real-time polymerase chain reaction (RT-PCR) for hepatic gene expression

Total RNA was extracted from the liver samples using TRIzol reagent (#15596018, Thermo Fisher Scientific, Waltham, MA, USA) (Ncho et al., 2023). The optical density of the obtained RNA was measured at 260 and 280 nm using a Nanodrop (Thermo Scientific, Waltham, MA, USA). The ratio of the densities thus obtained was used to determine the purity of the RNA. Next, a cDNA synthesis kit (# AB1453A, Thermo Fisher Scientific, Waltham, MA, USA) was used to synthesize cDNA from the hepatic RNA. The cDNA was used to quantify the mRNA expression levels of several genes (primer sequences in Table 1) via a StepOnePlus™ real-time PCR system (Life Technologies, Carlsbad, CA, USA). Each reaction consisted of 10 pmol of forward and reverse primers specific to the target genes, synthesized cDNA, and 10 µL of Power SYBR Green PCR Master Mix (#4312704, Life Technologies, Carlsbad, CA, USA). Two housekeeping genes, glyceraldehyde-3-phosphate dehydrogenase (GAPDH) and β-actin, were used to normalize the gene expression. Fold change was calculated using the 2–ΔΔCt algorithm as previously described (Livak and Schmittgen, 2001), while the regulation of gene expression was reported as log2 fold change, as described in previous studies (Pietrzak et al., 2020).

**Table 1:** Oligonucleotide primer sequence for RT-qPCR.

No.	Gene	Sequence	Accession Number	Reference
1.	GAPDH	F: TTGGCATTGTGGAGGGTCTTA R: GTGGACGCTGGGATGATGTT	NM_204305.1	(Goel et al., 2021)
2.	β-actin	F: ACCGGACTGTTACCAACA R: GACTGCTGCTGACACCTT	NM_205518.1	(Zhang et al., 2018)
3.	NRF2	F: CAGAAGCTTTCCCGTTCATAGA R: GACATTGGAGGGATGGCTTAT	NM_205117	(Zhang et al., 2018)
4.	CAT	F: ACCAAGTACTGCAAGGCGAA R: TGAGGGTTCCTCTTCTGGCT	NM_001031215.1	(Goel et al., 2021)
5.	SOD	F: AGGGGGTCATCCACTTCC R: CCCATTTGTGTTGTCTCCAA	NM_205064.1	(Goel et al., 2021)
6.	GPX1	F: AACCAATTCGGGCACCAG R: CCGTTCACCTCGCACTTCTC	NM_001277853.2	(Ncho et al., 2021b)
7.	NOX1	F: GCGAAGACGTGTTCCTGTAT R: GAACCTGTACCAGATGGACTTC	NM_001101830.1	(Ncho et al., 2023)
8.	NOX4	F: CCTCTGTGCTTGTACTGTGTAG R: GACATTGGAGGGATGGCTTAT	NM_001101829.1	(Ncho et al., 2023)
9.	DNMT1	F: ACAGCCTTCGCCGATTACA R: CTCTCCACCTGCTCCACCAC	NM_206952.1	(Zhu et al., 2020)
10.	DNMT 3A	F: GGATAGCCAAGTTCAGCAAAG R: GGGAAGCCAAACACCCTCT	NM_001024832.1	(Zhu et al., 2020)
11.	DNMT 3B	F: GTGCTGTGCCTTGAACATTG R: TTCGTAACTTCGGAAACCATT	NM_001024828.1	(Zhu et al., 2020)
12.	TET1	F: GGGACAACCGACTGACTCTG	XM_015278732.1	(Zhu et al., 2020)
		R: GAGATCCGCGTGGGATGATT		
13.	TET2	F: AGGCTATGGTGGTAGCCTCA R: GAGCAGCGTGCTTGTGAAAA	NM_001277794.1	(Zhu et al., 2020)
14.	TET3	F: ACAGGAACACGGATTTCCCC R: TTCTGTGCAAATGGCGATGC	XM_015297468.1	(Zhu et al., 2020)
15.	GADD45A	F: CTTGGCCCAGTTGTTGCTTC R: CCGGCACCCACTGATCCATA	DQ358721.1	(Zhu et al., 2019)
16.	TDG	F: GTTTCGAGAAGGAGGGCGAA R: CACGAGGGAACTGAGCACAT	NM_204750.1	(Zhu et al., 2020)
17.	MBD4	F: GGAAGTACCCCTCTCCCGAA R: GTGCAGCTCAATGGGGTACT	NM_204693.1	(Zhu et al., 2020)

Abbreviations: GAPDH, Glyceraldehyde-3-phosphate dehydrogenase; β-actin, Beta-actin; NRF2, Nuclear factor erythroid 2-related factor; CAT, Catalase; SOD, Superoxide dismutase; GPX1, Glutathione peroxidase 1; NOX1, Nicotinamide adenine dinucleotide phosphate oxidase 1; NOX4, Nicotinamide adenine dinucleotide phosphate oxidase 4; DNMT1, DNA methyltransferase 1; DNMT3A, DNA methyltransferase 3A; DNMT3B, DNA methyltransferase 3B; TET1, Ten-eleven translocation methylcytosine dioxygenase 1; TET2, Ten-eleven translocation methylcytosine dioxygenase 2; TET3, Ten-eleven translocation methylcytosine dioxygenase 3; GADD45A, Growth arrest and DNA damage-inducible proteins 45 alpha; TDG, Thymine DNA Glycosylase; MBD4, Methyl-CpG-binding domain protein 4.

### Statistical analysis

Each cage was considered as an experimental unit for the duration of the study. Hence, each treatment group had six replicates. During the experimental period, mortality was recorded to adjust the growth-related results accordingly. The assumptions for the parametric tests were assessed using Shapiro–Wilk and Levene’s tests. After the assumptions for normality of distribution and homoscedasticity were met, relevant parametric tests were applied.

Growth related data (BW initial, BW final, BW change, ADG, ADFI, and FCR), various organ weights (liver, spleen, bursa of Fabricius), plasma antioxidant capacity (DPPH-RSA%, MDA concentration, antioxidant balance), and rectal temperature (RT initial, RT final, and RT change) were analyzed using one-way analysis of variance (ANOVA) test. Tukey’s post-hoc test was used to indentify the significant differences (p < 0.05). Furthermore, linear and quadratic regression analysis (excluding the 0TAU-TN group) were also performed to study the dose-related effects of taurine. One-way ANOVA and polynomial regression were conducted using IBM SPSS Statistics for Windows software (IBM SPSS 27; IBM Corp., Armonk, NY, USA).

Further, various hepatic genes were categorized as antioxidant-related genes (NRF2, CAT, SOD, and GPX1), NOX-related genes (NOX1 and NOX4), DNA-methylation related genes (DNMT1, DNMT3A, and DNMT3B), DNA-demethylation related genes (TET1, TET2, TET3, TDG, MBD4, and GADD45A). These genes were analyzed using one-way multivariate analysis of variance (MANOVA) in the SAS software version 9.4 (SAS Institute Inc., Cary, NC, USA, 2009) using “manova statement” and a planned contrast analysis was performed to compare 0TAU-TN vs 0TAU-HS, 0TAU-HS vs 1TAU-HS, 0TAU-HS vs 3TAU-HS, and 0TAU-HS vs 5TAU-HS using a “contrast statement”.

To identify clustering patterns among hepatic genes and treatment groups, a hierarchically clustered heatmap was generated based on Euclidean distance. Further, to explore overall patterns and treatment effects in gene expression profiles, a Principal Component Analysis (PCA) was performed using normalized expression values of hepatic genes. To compare the relative levels of key plasma biochemical parameters across different treatment groups, a radar chart was generated. Finally, a Karl-Pearson’s correlation analysis was done to evaluate the co-regulation of the studied genes. The heat map, PCA, spider chart, and correlation map were obtained from R software version 4.0.3 (R Core Team, 2024) using “Complexheatmap”, “FactoMineR”, “fmsb”, and “corrplot” packages, respectively.

## Results

### Growth performances and organ indexes

Table 2 shows the initial body weight (BW), final BW, change in BW (%), average daily gain (ADG), average daily feed intake (ADFI), and feed conversion ratio (FCR) during the HS period. While the other parameters were not affected significantly, ADG increased linearly (p = 0.032) with the concentration of taurine.

**Table 2:** Effects of in ovo taurine feeding on growth of broilers under cyclic HS.

Parameters	Treatments					Pooled SEM	p-value		
	0TAU-TN	0TAU-HS	1TAU-HS	3TAU-HS	5TAU-HS		ANOVA	Lin*	Quad*
BW initial	1391.22	1426.97	1390.17	1441.75	1449.92	13.998	0.552	0.381	0.469
BW final	2012.42	2016.38	1996.42	2039.92	2110.42	22.442	0.552	0.140	0.354
BW change	44.61	41.26	43.70	41.49	45.45	0.578	0.065	0.074	0.549
ADG	88.74	84.20	86.61	85.45	95.29	1.521	0.151	0.032	0.246
ADFI	178.15	166.19	166.71	167.30	178.80	2.297	0.174	0.057	0.206
FCR	2.01	1.98	1.93	1.97	1.88	0.019	0.240	0.176	0.756

At 17.5 ED, eggs were injected with 0.6mL taurine solution with a concentration of 0 % (0TAU), 1.0 % (1TAU), 3.0 % (3TAU), and 5 % (5TAU). During rearing days 29 to 34, broilers were exposed to a cyclic heat stress (HS, 31 ± 1 °C, 8 hours) or kept at a thermoneutral temperature (TN) zone (21 ± 1 °C).

⁎ p value of all treatment groups except 0TAU-TN. The orthogonal tests were based on 0TAU through 5TAU under HS. Data are presented as Mean ± SEM (n = 6) Abbreviations: BW, body weight; ADG, average daily gain; ADFI, average daily feed intake; FCR, feed-conversion ratio; Lin, linear effect; Quad, quadratic effect.

The absolute and relative weights of immune organs such as liver, spleen and bursa of Fabricius have been reported in Table 3, and show no significant differences across the treatments.

**Table 3:** Effects of in ovo taurine feeding on organ indices of broilers under cyclic HS.

Parameters	Treatments					Pooled SEM	p-value		
	0TAU-TN	0TAU-HS	1TAU-HS	3TAU-HS	5TAU-HS		ANOVA	Lin*	Quad*
Absolute weight									
Liver	50.95	58.55	61.72	52.38	60.04	1.752	0.202	0.778	0.569
Spleen	1.87	2.67	2.30	2.57	3.41	0.220	0.268	0.292	0.253
Bursa	3.39	3.81	2.81	3.84	3.37	0.173	0.338	0.862	0.521
Relative weight (g/100gBW)									
Liver	2.40	2.91	2.91	2.54	2.80	0.074	0.090	0.353	0.444
Spleen	0.09	0.13	0.11	0.13	0.16	0.010	0.296	0.433	0.246
Bursa	0.16	0.19	0.13	0.18	0.16	0.008	0.216	0.630	0.431

At 17.5 ED, eggs were injected with 0.6mL taurine solution with a concentration of 0 % (0TAU), 1.0 % (1TAU), 3.0 % (3TAU), and 5 % (5TAU). During rearing days 29 to 34, broilers were exposed to a cyclic heat stress (HS, 31 ± 1 °C, 8 hours) or kept at a thermoneutral temperature (TN) zone (21 ± 1 °C).

⁎ p value of all treatment groups except 0TAU-TN. The orthogonal tests were based on 0TAU through 5TAU under HS. Data are presented as Mean ± SEM (n = 6).

Table 4 shows the absolute and relative lengths of various parts of the small intestine (duodenum, jejunum, and ileum) and average cecal length, which did not vary significantly across the treatments.

**Table 4:** Effects of in ovo taurine feeding on intestinal length indices of broilers under cyclic HS.

Parameters	Treatments					Pooled SEM	p-value		
	0TAU-TN	0TAU-HS	1TAU-HS	3TAU-HS	5TAU-HS		ANOVA	Lin*	Quad*
Absolute length (cm)									
Duodenum	29.17	30.58	28.67	28.58	28.67	0.414	0.534	0.171	0.288
Jejunum	68.00	71.25	70.25	72.33	74.33	1.131	0.506	0.280	0.525
Ileum	68.00	71.42	70.67	69.58	68.25	1.207	0.893	0.369	0.913
Cecum#	17.08	15.96	17.63	17.71	17.71	0.300	0.297	0.078	0.204
Relative length (cm/100gBW)									
Duodenum	1.39	1.53	1.35	1.38	1.35	0.030	0.318	0.122	0.311
Jejunum	3.24	3.54	3.30	3.51	3.51	0.069	0.546	0.852	0.462
Ileum	3.24	3.54	3.33	3.38	3.21	0.103	0.585	0.183	0.876
Cecum#	0.81	0.79	0.83	0.86	0.84	0.019	0.851	0.430	0.522

At 17.5 ED, eggs were injected with 0.6mL taurine solution with a concentration of 0 % (0TAU), 1.0 % (1TAU), 3.0 % (3TAU), and 5 % (5TAU). During rearing days 29 to 34, broilers were exposed to a cyclic heat stress (HS, 31 ± 1 °C, 8 hours) or kept at a thermoneutral temperature (TN) zone (21 ± 1 °C).

⁎ p value of all treatment groups except 0TAU-TN. The orthogonal tests were based on 0TAU through 5TAU under HS. Data are presented as Mean ± SEM (n = 6).

### Rectal temperature

The RT of the birds, recorded as initial (before HS) and final (after the end of HS), is displayed in Table 5. While the initial RT was not significantly different across the treatments, the final RT was significantly higher in the 5TAU-HS group compared to 0TAU-TN (p = 0.032). Further, the change in RT was significantly higher in the 0TAU-HS, 1TAU-HS, and 5TAU-HS compared to 0TAU-NT group (p = 0.003)

**Table 5:** Effect of in ovo taurine feeding on the rectal temperature of broilers under HS.

Parameters	Treatments					Pooled SEM	p-value		
	0TAU-TN	0TAU-HS	1TAU-HS	3TAU-HS	5TAU-HS		ANOVA	Lin*	Quad*
RT initial (°C)	40.90	40.03	40.00	40.70	41.17	0.208	0.292	0.052	0.587
RT final (°C)	42.13^a^	43.80^ab^	43.03^ab^	43.13^ab^	44.33^b^	0.239	0.032	0.473	0.057
RT change (°C)	1.23^a^	3.77^b^	3.03^b^	2.43^ab^	3.17^b^	0.234	0.003	0.240	0.099

At 17.5 ED, eggs were injected with 0.6mL taurine solution with a concentration of 0 % (0TAU), 1.0 % (1TAU), 3.0 % (3TAU), and 5 % (5TAU). During rearing days 29 to 34, broilers were exposed to a cyclic heat stress (HS, 31 ± 1 °C, 8 hours) or kept at a thermoneutral temperature (TN) zone (21 ± 1 °C).

⁎ p value of all treatment groups except 0TAU-TN. The orthogonal tests were based on 0TAU through 5TAU under HS. Data are presented as Mean ± SEM (n = 6).

a-b : Means with different letters indicate significant differences (p < 0.05).

### Plasma antioxidant capacity

The total free radical scavenging activity of plasma was measured via DPPH-RSA (%), showing that 0TAU-TN, 3TAU-HS and 5TAU-HS had significantly higher radical scavenging ability compared to 0TAU-HS and 1TAU-HS (Table 6). It was seen to further have a dose response where the DPPH-RSA% showed a linear increase (p = 0.001). Furthermore, the concentration of the lipid peroxidation product (MDA) was significantly higher in 0TAU-HS, 1TAU-HS, and 5TAU-HS compared to 0TAU-TN (p = 0.001). The MDA concentration showed a quadratic effect when the dose of taurine was increased (p = 0.005). Finally, the antioxidant balance was calculated, which was significantly lower in all the treatments under HS as compared to 0TAU-TN.

**Table 6:** Effect of in ovo taurine feeding on the antioxidant capacity and metabolites concentration in plasma of broilers under HS.

Antioxidant parameters	Treatments					Pooled SEM	p-value		
	0TAU-TN	0TAU-HS	1TAU-HS	3TAU-HS	5TAU-HS		ANOVA	Lin*	Quad*
DPPH-RSA (%)	19.85^b^	11.69^a^	14.07^a^	19.06^b^	19.98^b^	0.735	0.001	0.001	0.431
MDA (nmol/mL)	0.52^a^	1.08^b^^^c^^	1.06^b^^^c^^	0.78^ab^	1.39^c^	0.075	0.001	0.216	0.005
Antioxidant balance	58.36^a^	10.95^b^	13.58^b^	28.34^b^	14.40^b^	3.951	0.001	0.159	0.028
Metabolites concentration									
Glucose (mg/dL)	269.50	277.83	328.83	263.83	320.83	9.702	0.085	0.546	0.509
Total protein (g/dL)	3.10	3.35	3.67	3.07	3.75	0.110	0.152	0.623	0.509
Albumin (g/dL)	1.20	1.30	1.35	1.22	1.47	0.037	0.131	0.364	0.268
Globulin (g/dL)	1.90	2.03	2.18	1.87	2.33	0.072	0.211	0.471	0.385
Albumin: Globulin	0.63	0.63	0.58	0.63	0.65	0.011	0.356	0.379	0.187
ALT (U/L)	17.67^ab^	14.33^ab^	12.50^a^	12.33^a^	23.50^b^	1.299	0.022	0.021	0.005
AST (U/L)	394.67	301.83	341.67	268.33	395.00	20.172	0.182	0.221	0.244
Cholesterol (mg/dL)	120.67	141.50	151.17	132.83	156.83	4.855	0.123	0.579	0.526
Triglycerides (mg/dL)	63.00	82.17	86.67	86.00	85.50	4.163	0.339	0.824	0.495

At 17.5 ED, eggs were injected with 0.6mL taurine solution with a concentration of 0 % (0TAU), 1.0 % (1TAU), 3.0 % (3TAU), and 5 % (5TAU). During rearing days 29 to 34, broilers were exposed to a cyclic heat stress (HS, 31 ± 1 °C, 8 hours) or kept at a thermoneutral temperature (TN) zone (21 ± 1 °C).

⁎ p value of all treatment groups except 0TAU-TN. The orthogonal tests were based on 0TAU through 5TAU under HS. Data are presented as Mean ± SEM (n = 6).

a-b : Means with different letters indicate significant differences (p < 0.05). Abbreviations: DPPH-RSA ( %), 2,2-diphenyl-1-picrylhydrazyl free radical scavenging activity; MDA, malondialdehyde; AST, aspartate aminotransferase; ALT, alanine transaminase.

### Plasma metabolites

**Fig. 2:**
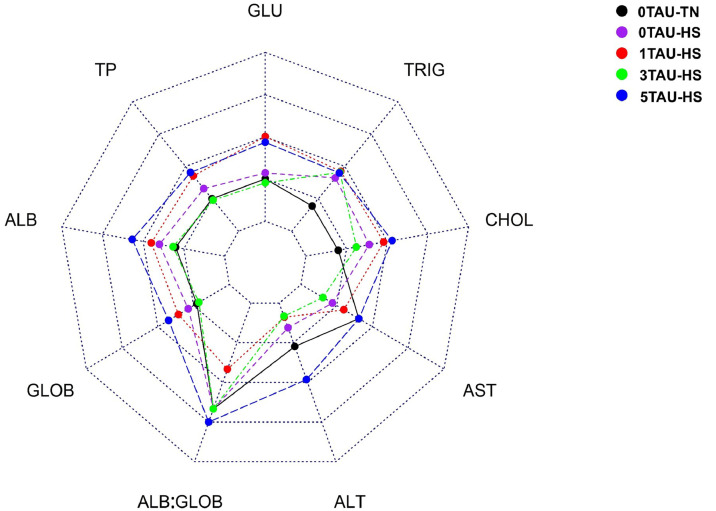
Spider chart comparing plasma profile of broilers under HS. Each axis represents a different metabolite, while different colored lines indicate different treatments (0TAU-TN: black; 0TAU-HS: purple; 1TAU-HS: red; 3TAU-HS: green; 5TAU-HS: blue).

Abbreviations: GLU, glucose; TRIG, triglycerides; CHOL, cholesterol; AST, aspartate aminotransferase; ALT, alanine transaminase; ALB.GLOB: albumin/globulin ratio; GLOB: globulin; ALB: albumin; TP, total protein.

Table 6 demonstrates the various metabolites studied in plasma. Among the study parameters, only alanine transaminase (ALT) was significantly affected, with lower levels in the 1TAU-HS and 3TAU-HS groups compared to the 5TAU-HS group (p = 0.022). Further, a spider chart was created to observe the overall plasma metabolic profile of the birds (Fig. 2). This revealed that various metabolites were higher in the 5TAU-HS group compared to the 0TAU-TN group.

### Hepatic gene expression of various genes

**Fig. 3:**
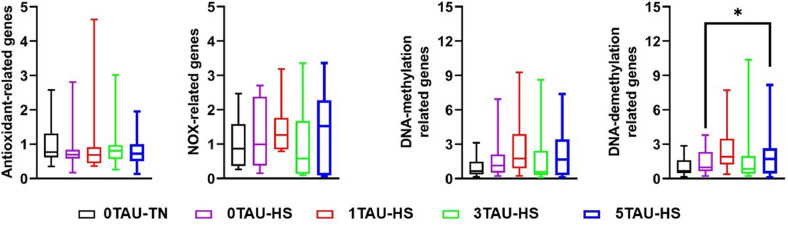
Boxplot showing the effect of in ovo taurine feeding on the hepatic mRNA gene regulation of several gene groups such as: antioxidant-related, NOX-related, DNA-methylation related, and DNA-demethylation related. Data are presented as Mean ± SEM. *Means with different symbols indicate significant differences.

The hepatic mRNA expression of the studied genes did not vary significantly across treatments (Supplementary Table 2). Further, when several genes related to a common function, such as antioxidant-related (NRF2, SOD, CAT, GPX1), NOV-related (NOX1 and NOX4), DNA-demethylation related (TET1, TET2, TET3, TDG, MBD4, and GADD45A), and DNA-methylation related (DNMT1, DNMT3A, and DNMT3B), were subjected to MANOVA (Fig. 3). The MANOVA did not reveal any significant differences, whereas a planned contrast (Table 7) between the 5TAU-HS and 0TAU-TN groups showed that DNA-methylation-related genes were highly expressed in the 5TAU-HS group (p = 0.030).

**Table 7:** MANOVA and multivariate planned contrasts on different sets of hepatic gene expressions of broilers subjected to HS.

Set of genes	MANOVA	Multivariate Planned Contrast			
		0TAU-TN vs 0TAU-HS	0TAU-HS vs 1TAU-HS	0TAU-HS vs 3TAU-HS	0TAU-HS vs 5TAU-HS
Antioxidant-related genes	0.634	0.377	0.642	0.986	0.890
NOX-related genes	0.804	0.829	0.591	0.436	0.582
DNA-demethylation related genes	0.104	0.093	0.476	0.340	0.030
DNA-methylation related genes	0.458	0.431	0.344	0.521	0.232

At 17.5 ED, eggs were injected with 0.6mL taurine solution with a concentration of 0 % (0TAU), 1.0 % (1TAU), 3.0 % (3TAU), and 5 % (5TAU). During rearing days 29 to 34, broilers were exposed to a cyclic heat stress (HS, 31 ± 1 °C, 8 hours) or kept at a thermoneutral temperature (TN) zone (21 ± 1 °C).

### Interactions and relationships among various hepatic genes studied

**Fig. 4:**
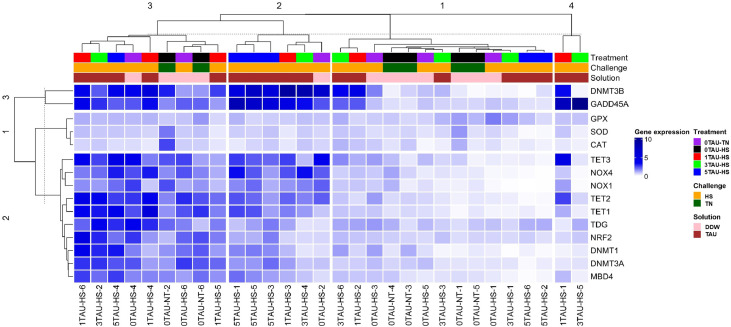
Hierarchically clustered heat map of hepatic gene expression of various genes studied. Each column represents an experimental unit, while each row represents a specific gene. RT-qPCR was used for gene expression analysis. GAPDH and β-actin were used as reference genes, and the fold change (FC) of the genes was calculated as 2^−ΔΔCt^. The relative gene expression values were obtained as log_2_(FC). The tree was constructed using the package “ComplexHeatmap” of the R software version 4.0.3 (R Core Team, 2020). Abbreviations: Dim, Dimension; DDW, Distilled water, TAU, Taurine; GAPDH, Glyceraldehyde-3-phosphate dehydrogenase; β-actin, Beta-actin; NRF2, Nuclear factor erythroid 2-related factor; CAT, Catalase; SOD, Superoxide dismutase; GPX1, Glutathione peroxidase 1; NOX1, Nicotinamide adenine dinucleotide phosphate oxidase 1; NOX4, Nicotinamide adenine dinucleotide phosphate oxidase 4; DNMT1, DNA methyltransferase 1; DNMT3A, DNA methyltransferase 3A; DNMT3B, DNA methyltransferase 3B; TET1, Ten-eleven translocation methylcytosine dioxygenase 1; TET2, Ten-eleven translocation methylcytosine dioxygenase 2; TET3, Ten-eleven translocation methylcytosine dioxygenase 3; GADD45A, Growth arrest and DNA damage-inducible proteins 45 alpha; TDG, Thymine DNA Glycosylase; MBD4, Methyl-CpG-binding domain protein 4.

A hierarchically clustered heatmap based on Euclidean distances has been displayed as Fig. 4. Each vertical stack represents an individual bird and islabelled at the bottom, while each horizontally stacked square represents one specific gene which was studied. In the clustering, the heatmap was divided into four vertical and 3 horizontal clusters. In the horizontal clusters, antioxidant genes such as GPX1, SOD, and CAT formed cluster 1, indicating a fair degree of co-regulation among these genes. The second horizontal cluster consisted of all the other genes except the DNMT3B and GADD45A, which formed the third horizontal cluster. Next, the first vertical cluster was majorly dominated by individuals fed DDW during incubation and subjected to HS. This cluster denoted a low expression of almost all the studied genes. Cluster 2, on the other hand, mostly denoted the birds which were injected with taurine and subjected to HS. This cluster exhibited lower expression of antioxidant enzymes but higher expression of the DMT3B and GADD45A genes. The third vertical cluster comprised individuals fed with DDW and taurine, but mostly subjected to HS. The birds in cluster 3 exhibited a higher expression of all genes, except for those related to antioxidants. Finally, the smallest cluster was formed only by two birds, which were injected with in ovo taurine and subjected to HS. This cluster exhibited low expression of almost all genes, except for DNMT3B and GADD45A.

**Fig. 5:**
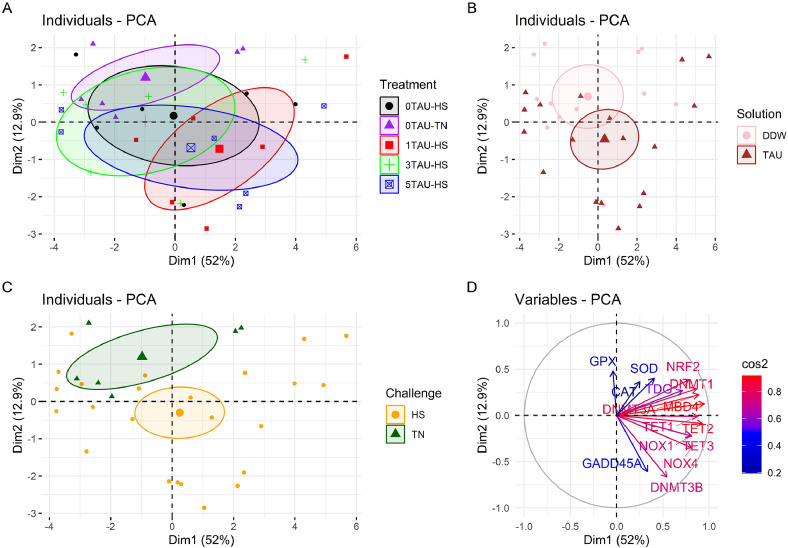
Principal component analysis (PCA) plot of individuals and variables. An individual refers to a sampled bird per treatment, while a variable is a biological parameter analysed. Different colors have been allotted to various treatments (A), solutions (B), challenge (C), and finally, various variables based on their squared cosines (D).This figure was constructed using “FactoMineR” package of the R software version 4.0.3 (R Core Team, 2020). Abbreviations: Dim, Dimension; DDW, Distilled water, TAU, Taurine; NRF2, Nuclear factor erythroid 2-related factor; CAT, Catalase; SOD, Superoxide dismutase; GPX1, Glutathione peroxidase 1; NOX1, Nicotinamide adenine dinucleotide phosphate oxidase 1; NOX4, Nicotinamide adenine dinucleotide phosphate oxidase 4; DNMT1, DNA methyltransferase 1; DNMT3A, DNA methyltransferase 3A; DNMT3B, DNA methyltransferase 3B; TET1, Ten-eleven translocation methylcytosine dioxygenase 1; TET2, Ten-eleven translocation methylcytosine dioxygenase 2; TET3, Ten-eleven translocation methylcytosine dioxygenase 3; GADD45A, Growth arrest and DNA damage-inducible proteins 45 alpha; TDG, Thymine DNA Glycosylase; MBD4, Methyl-CpG-binding domain protein 4.

Furthermore, the individuals were projected onto two dimensions based on hepatic gene expression data, and a PCA plot was generated (Fig. 5). The two dimensions accounted for 64.9 % of the dataset's variability (Dim 1: 52 % + Dim 2: 12.9 %). The plot was divided into four subparts, each based on individuals and variables studied. A differential color scheme was chosen to demonstrate the differences based on various treatments (Fig. 5A), solutions (Fig. 5B), and challenges (Fig. 5C). Lastly, a variables graph was created based on the squared cosines (Fig. 5D). Interestingly, almost all the genes were found to be related to dimension 1, except for GPX1, CAT and GADD45A, which showed inclination towards dimension 2. While the treatment PCA (Fig. 5A) showed several overlapping circles, the solutions PCA (Fig. 5B) displayed a slight overlap between the DDW and taurine-injected individuals. While most of the taurine-injected birds were centered towards the lower two quartiles of the graph (brown colored), the DDW-injected individuals (pink colored) were more inclined towards the upper left quartile. Further, HS and TN birds showed a clear distinction, wherein the HS birds clustered around the origin, while the TN birds were more focused on the upper left quartile of the graph.

**Fig. 6:**
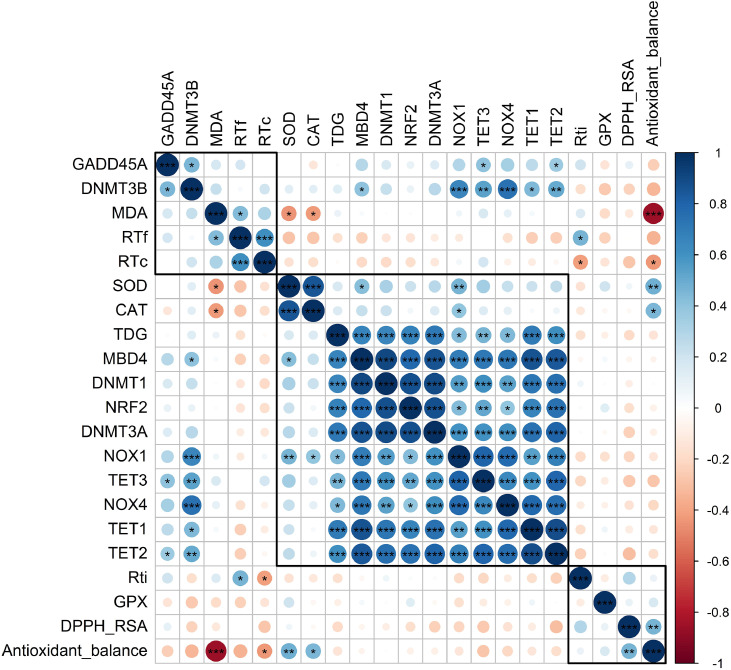
Pearson’s correlation heat map among various hepatic mRNA gene expressions, plasma antioxidant capacity, and rectal temperature of broilers under HS. The matrix is visualized based on hierarchical clustering of correlated data. Blue and red colors indicate positive and negative correlations, respectively, with intensity proportional to the strength of correlation (r). The graph was constructed using the package “corrplot” of the R software version 4.0.3 (R Core Team, 2020). * indicates significant correlation at p < 0.05 level. ** indicates significant correlation at p < 0.01 level. *** indicates significant correlation at p < 0.001 level.

Abbreviations: NRF2, Nuclear factor erythroid 2-related factor; CAT, Catalase; SOD, Superoxide dismutase; GPX1, Glutathione peroxidase 1; NOX1, Nicotinamide adenine dinucleotide phosphate oxidase 1; NOX4, Nicotinamide adenine dinucleotide phosphate oxidase 4; DNMT1, DNA methyltransferase 1; DNMT3A, DNA methyltransferase 3A; DNMT3B, DNA methyltransferase 3B; TET1, Ten-eleven translocation methylcytosine dioxygenase 1; TET2, Ten-eleven translocation methylcytosine dioxygenase 2; TET3, Ten-eleven translocation methylcytosine dioxygenase 3; GADD45A, Growth arrest and DNA damage-inducible proteins 45 alpha; TDG, Thymine DNA Glycosylase; MBD4, Methyl-CpG-binding domain protein 4; RTi, rectal temperature initial; RTf, rectal temperature final; RTc, change in rectal temperature; DPPH_RSA, 2,2-diphenyl-1-picrylhydrazyl free radical scavenging activity; MDA, malondialdehyde.

Lastly, to investigate the relationships among oxidative stress markers, antioxidant enzymes, epigenetic regulators, and antioxidant balance, a Pearson correlation analysis was performed in R software and visualized as a correlation matrix (Fig. 6). The matrix displays the direction (white to blue: positive and white to red: negative) and strength of the correlation between variables. The magnitude of the correlations has been indicated by the size and color of the circles, and statistical significance is annotated using asterisks (*: p < 0.05; **: p < 0.01; and ***: p < 0.001). The most notable positive interactions can be visualized in the center cluster, which includes DNA methylation-related genes (DNMT1 and DNMT3A), DNA demethylation-related genes (TET1, TET2, TET3, TDG, and MBD4), and NOX-related genes (NOX1 and NOX4), suggesting a coordinated regulation of these pathways. The MDA content of plasma was negatively correlated with antioxidant genes, such as SOD and CAT.

## Discussion

HS-induced growth impediment is evidenced in several studies. It was shown that the final BW, BWG, FI, and feed efficiency of broiler chickens under HS were significantly reduced (Goo et al., 2019). Under chronic HS conditions, studies have reported a decline in BW (Sohail et al., 2012). Supplementation of taurine at varied levels in broiler chickens and turkeys did not significantly affect their growth (Blair et al., 1991; Tufft and Jensen, 1992). However, under chronic HS conditions, dietary supplementation of taurine at 0.1 % helped recover the BW loss compared with control in broiler chickens (Belal et al., 2018). Further, while HS reduced BWG in broiler chickens, supplementation of 0.8 % taurine in the diet improved BWG compared to the HS group (Shim et al., 2006). The current study shows that taurine linearly improved ADG during HS (p = 0.032), suggesting enhanced nutrient metabolism and resilience under thermal stress. Under general physiological “stress-free” conditions, taurine is biosynthesized in the body by utilizing dietary taurine and is therefore categorized as a non-essential amino acid (Miraglia et al., 1966). Although, as the number of days to reach a marketable weight in broiler chickens decreases rapidly, the susceptibility to various stressors increases (Riber and Wurtz, 2024). This may warrant a reconsideration of the “essentiality” of certain nutrients. Furthermore, changes in BW and ADFI showed a linear increasing trend (0.05 < p < 0.10) as the concentration of in ovo taurine was increased. This can be correlated with the linearly increasing ADG. Summarising the growth results of this study and comparing with the previous studies, the mode and frequency of supplementation could be one of the major factors determining the final effects. When supplemented in diet, a daily dose of taurine might have superior benefits on growth.

Interestingly, no significant differences in various plasma metabolite levels were observed between the groups, regardless of whether or not they were exposed to HS, except for the concentration of alanine aminotransferase (ALT). Primarily found in the cytoplasm of hepatocytes, ALT is one of the primary enzymes released by a damaged liver into the bloodstream (Moriles et al., 2024). Elevated ALT levels are often a sign of liver injury (Senior, 2012). However, it has been shown that elevated ALT in the bloodstream may also be due to skeletal muscle injury (Bardella et al., 1999). Previous studies have shown that dietary supplementation of taurine at 300 to 900 mg/Kg of diet decreased serum ALT and MDA concentration in 42-day-old broiler chickens raised under summer conditions (Abd Al-Qaisy and Saied, 2023). In sodium fluoride-induced toxicity models in chickens, supplementing taurine at 3g/Kg of diet alleviated hepatic injury. This was demonstrated by lower liver function enzyme concentrations (AST and ALT) and MDA content (Alabsy and Alabdaly, 2022). The hepato-protective effect of taurine was attributed to its antioxidant and anti-inflammatory properties (Ji et al., 2023). However, in the current study, 5TAU-HS group had the highest plasma ALT concentration compared to the 1TAU-HS and 3TAU-HS groups. Conversely, the 5TAU-HS group also had the highest plasma MDA concentration compared to 0TAU-TN and 3TAU-HS groups. Hence, a striking hepato-protective effect of in ovo taurine was not appreciated. One of the reasons for this result could be no fasting period for the chickens before slaughter. Hence, further studies are warranted in this direction to ascertain the plausible mechanism of the results obtained.

Under HS, oxidative stress follows, which causes a surge in the release of free radicals. These free radicals, such as reactive oxygen species (ROS) and reactive nitrogen species (RNS), induce cellular membrane damage and produce metabolites such as MDA (Wang et al., 2020). Under stress conditions, the production of these free radicals is increased manifold, leading to extensive cellular damage (Aryal et al., 2025). Various organs, such as the liver, may be negatively affected by excessive ROS production, leading to injury and hepatocytes damage. However, it was reported that lipid peroxidation was significantly reduced (as indicated by lower MDA content) in chicks supplemented with 0.8 % taurine under HS (Shim et al., 2006). In contrast, the current study shows that MDA content was higher in all HS groups compared to TN, except in the 3TAU-HS group. The current results of plasma MDA concentration and ALT levels indicate hepatic membrane injury and damage to hepatocytes in 5TAU-HS treatment. The discrepancy in the results from previous reports might be attributed to differences in mode, dose, and supplementation period. It has been reported that the volume and concentration of the substance fed highly governs the outcome of in ovo feeding (Uni et al., 2005). Hence, in ovo feeding of 5 % taurine might cause hepato-injury. Hence, further studies with different doses are needed to underline the effects of in ovo taurine on broiler chickens under HS. As discussed before, HS leads disrupts the balance between antioxidant defense mechanism and ROS/RNS species, leading to a state of oxidative stress (Oke et al., 2024). The primary antioxidant defense enzymes are Glutathione peroxidase 1 (GPX1), catalase (CAT), and superoxide dismutase (SOD), which are responsible for eradicating the free radicals formed (Mehta and Gowder, 2015). This enzymatic machinery is superheaded by NRF2 (Ma, 2013). Taurine is a known antioxidant (Surai et al., 2021). Therefore, in this study, we investigated the effect of in ovo taurine on the hepatic mRNA expression of these enzymes. It was reported that dietary supplementation of taurine at 5g/Kg feed activated the NRF2 pathway and alleviated the adverse effects on breast muscle quality in 28-day-old broiler chickens under chronic HS (Lu et al., 2019b). Another study encompassing dietary supplementation at varied levels of taurine (0.25, 5.00, and 7.70g/Kg diet) showed that taurine at 5g/Kg diet increased the hepatic expression of GPX and NRF2 in male Arbor Acre broiler chickens (Han et al., 2020b). Studies have demonstrated the antioxidant properties of taurine against several oxidative stress-inducing agents, such as lipopolysaccharides (Han et al., 2020a), isoprenaline (Ohta et al., 1986), doxorubicin (Hamaguchi et al., 1989), and ethanol (Berlin et al., 2010). However, the current study showed no significant differences in the hepatic gene expression of several antioxidant enzymes studied. While the exact mechanism is unclear, we suggest that dietary supplementation may yield superior results due to variations in the amount and duration of supplementation. Also, the differences in the form and duration of stress might have played a role in determining the results. Hence, further studies, possibly comparing in ovo feeding methods with dietary supplementation at different doses, could give better comprehension.

In the current study, while the majority of the genes remained significantly unaffected, a planned contrast revealed a higher expression of DNA-demethylation-related genes in 5TAU-HS compared to 0TAU-HS (p = 0.030). Unlike vitamin C (Blaschke et al., 2013) and alpha-ketoglutarate (Carey et al., 2015), taurine has not been well studied for its effects on DNA demethylation. However, the role of taurine in one-carbon metabolism via cysteine pathway could alter the S-adenosylmethionine (SAM)/S-adenosylhomocysteine (SAH) ratio (Zhang, 2018). In blood macrophages, taurine reduced the availability of SAM (Meng et al., 2021), which could potentially lower DNA-methylation levels. The epigenetic potential of taurine might also be attributed to a reduction in the formation of hydroxylated DNA bases, such as 5-OH-uracil and 8-OH-adenine, which are markers of oxidative DNA damage (Messina and Dawson Jr, 2002). Furthermore, taurine has the ability to scavenge free radicals, thereby protecting DNA from oxidative injury (Baliou et al., 2021). Strong positive correlations were observed among DNA methylation (DNMTs), demethylation (TETs), and NOX-related genes, implying coordinated regulation. Negative correlations between MDA and antioxidant enzymes (SOD, CAT) indicated oxidative stress-related damage under HS. Nevertheless, in the current study, the strong antioxidant role of in ovo taurine in broiler chickens under HS was not observed. Although the 5TAU-HS group showed an overall upregulation of DNA demethylation-related genes, the molecular pathways involved remain unknown. Further studies encompassing taurine supplementation and fetal epigenetic programming could provide valuable insights.

## Conclusion

Overall, in ovo taurine had marginal effects on broiler chickens under HS in terms of growth, antioxidant capacity, and hepatic gene expression of various enzymes. While strong epigenetic regulation was not appreciated, but in ovo feeding of 5 % taurine increased the expression of DNA-demethylation related genes. It is speculated that dietary supplementation could bear superior advantages.

## Funding

This work was partially supported by the Ministry of Science and ICT (MIST) through the National Research Foundation (NRF) of the Korean government (No. RS-2024-00350745) and additionally funded by the Brain Pool (BP) Program through the NRF grant (No. 2019H1D3A1A01071142) provided by the MIST.

## CRediT authorship contribution statement

**Vaishali Gupta:** Writing – review & editing, Writing – original draft, Software, Methodology, Investigation, Formal analysis, Data curation, Conceptualization. **Yun-Ji Hwang:** Writing – review & editing, Methodology, Investigation, Data curation. **Meg Aui Delmonte:** Writing – review & editing. **Chris Major Ncho:** Writing – review & editing. **Yang-Ho Choi:** Writing – review & editing, Resources, Conceptualization.

## Disclosures

The authors declare that they have no known competing financial interests or personal relationships that could have appeared to influence the work reported in this paper.

## Appendix Supplementary materials

Image, application 1

## Footnotes

Supplementary material associated with this article can be found, in the online version, at doi:10.1016/j.psj.2026.106450.
