# Supplementary material for: Effects of in ovo taurine administration on cyclic heat stress in broiler chickens
Source: Poult Sci. 2026 Jan 14;105(4):106450. doi: 10.1016/j.psj.2026.106450 (PMC12887376; doi:10.1016/j.psj.2026.106450)
Supplement: Supplementary file 1 [file mmc1.docx]

Supplementary Table 1. Feed composition and nutrient levels of the diet^*^

| Ingredients (%) | Phase I | Phase II |
| --- | --- | --- |
|  | 0-21 days | 22-35 days |
| Corn | 45.87 | 39.68 |
| Wheat | 15 | 25 |
| Soybean meal (42.6% CP) | 25.6 | 20.6 |
| Corn gluten | 2.64 | 3 |
| Meat and bone meal | 2 | 2.5 |
| Animal fat | 3.88 | 4.54 |
| Salt | 0.25 | 0.25 |
| Tricalcium phosphate | 1.04 | 0.86 |
| Limestone | 1.22 | 1.26 |
| Sodium bicarbonate | 0.02 | 0 |
| L-Threonine | 0.16 | 0.16 |
| Lysine | 1.44 | 1.32 |
| D-L-Methionine | 0.03 | 0.03 |
| Choline chloride (50%) | 0.03 | 0.03 |
| Premix^#^ | 0.2 | 0.2 |
| Phytase | 0.05 | 0.05 |
| Feed additive | 0.25 | 0.25 |
| Anti-coccidia | 0.01 | 0.01 |
| Calculated nutrients |  |  |
| Crude protein (%) | 20.5 | 19.5 |
| Crude fat (%) | 6.36 | 6.9 |
| Crude fibre (%) | 2.8 | 2.68 |
| Crude ash (%) | 5.34 | 5.02 |
| Calcium (%) | 0.9 | 0.86 |
| Available phosphorous (%) | 0.53 | 0.49 |
| Digestible lysine (%) | 1.24 | 1.09 |
| Digestible methionine + cystine (%) | 0.95 | 0.86 |
| Copper (ppm) | 81.04 | 80.78 |
| Zinc (ppm) | 96.63 | 97.33 |
| Metabolizable energy (kcal/kg) | 3150 | 3200 |

* Feed was procured from Nonghyup Feed (Seoul, Korea).

^#^ Trace minerals and vitamins provided in premix: Vitamin A, 12,000,000 IU; Vitamin D_3,_ 3,000,000 IU; Vitamin E, 40,000 IU; Vitamin K_3,_ 2,000 IU; Vitamin B_1,_ 2,000 mg; Vitamin B_2,_ 5,000 mg; Vitamin B_6,_ 3,000 mg; Vitamin B_12,_ 20 mg; Niacin, 40,000 mg; Pantothenic acid, 10,000 mg; Folic acid, 1,000 mg; Iron, 88,000 mg; Copper, 72,600 mg; Zinc, 60,000 mg; Manganese, 66,000 mg; Iodine, 990 mg; Selenium, 220 mg; Cobalt, 330 mg

Supplementary Table 2. Effect of in ovo taurine on hepatic gene expression of broilers under HS

| Genes studied | Treatments | | | | | p-value | | |
| --- | --- | --- | --- | --- | --- | --- | --- | --- |
|  | 0TAU-NT | 0TAU-HS | 1TAU-HS | 3TAU-HS | 5TAU-HS | ANOVA | Lin* | Quad* |
| NRF2 | 1.00 ± 0.30 | 1.27 ± 0.40 | 1.66 ± 0.63 | 1.14 ± 0.38 | 1.20 ± 0.23 | 0.833 | 0.703 | 0.712 |
| SOD | 1.00 ± 0.23 | 0.63 ± 0.05 | 0.66 ± 0.10 | 0.62 ± 0.09 | 0.56 ± 0.13 | 0.193 | 0.603 | 0.668 |
| CAT | 1.00 ± 0.33 | 0.55 ± 0.09 | 0.50 ± 0.08 | 0.58 ± 0.14 | 0.49 ± 0.08 | 0.234 | 0.766 | 0.845 |
| GPX1 | 1.00 ± 0.14 | 0.99 ± 0.17 | 0.83 ± 0.09 | 1.04 ± 0.08 | 0.82 ± 0.09 | 0.586 | 0.601 | 0.783 |
| NOX1 | 1.00 ± 0.36 | 1.25 ± 0.41 | 1.17 ± 0.15 | 0.74 ± 0.29 | 1.18 ± 0.38 | 0.818 | 0.648 | 0.424 |
| NOX4 | 1.00 ± 0.25 | 1.40 ± 0.44 | 1.72 ± 0.38 | 1.15 ± 0.53 | 1.74 ± 0.59 | 0.716 | 0.841 | 0.785 |
| TET1 | 1.00 ± 0.42 | 1.21 ± 0.40 | 2.44 ± 0.75 | 1.07 ± 0.47 | 1.81 ± 0.61 | 0.312 | 0.87 | 0.685 |
| TET2 | 1.00 ± 0.35 | 1.72 ± 0.45 | 2.55 ± 0.54 | 1.25 ± 0.51 | 1.47 ± 0.46 | 0.198 | 0.369 | 0.553 |
| TET3 | 1.00 ± 0.32 | 2.11 ± 0.58 | 2.50 ± 0.56 | 1.09 ± 0.36 | 1.99 ± 0.65 | 0.185 | 0.49 | 0.653 |
| TDG | 1.00 ± 0.24 | 1.52 ± 0.45 | 1.64 ± 0.36 | 1.29 ± 0.48 | 1.54 ± 0.31 | 0.761 | 0.865 | 0.873 |
| MBD4 | 1.00 ± 0.27 | 0.85 ± 0.22 | 1.47 ± 0.28 | 0.79 ± 0.28 | 1.33 ± 0.42 | 0.442 | 0.602 | 0.907 |
| GADD45A | 1.00 ± 0.29 | 1.60 ± 0.32 | 3.90 ± 0.90 | 3.37 ± 1.47 | 3.98 ± 1.38 | 0.147 | 0.191 | 0.453 |
| DNMT1 | 1.00 ± 0.33 | 1.07 ± 0.30 | 1.37 ± 0.55 | 0.91 ± 0.46 | 1.47 ± 0.47 | 0.863 | 0.714 | 0.774 |
| DNMT3A | 1.00 ± 0.31 | 1.40 ± 0.38 | 1.47 ± 0.37 | 0.95 ± 0.38 | 1.30 ± 0.37 | 0.789 | 0.625 | 0.708 |
| DNMT3B | 1.00 ± 0.46 | 2.46 ± 1.00 | 4.89 ± 0.97 | 2.64 ± 1.31 | 3.75 ± 1.18 | 0.121 | 0.754 | 0.577 |

*: p values of all the treatments except 0TAU-NT.

Abbreviations: NRF2, Nuclear factor erythroid 2-related factor; CAT, Catalase; SOD, Superoxide dismutase; GPX1, Glutathione peroxidase 1; NOX1, NADPH oxidase 1; NOX4, NADPH oxidase 4; TET1, Ten-eleven translocation methylcytosine dioxygenase 1; TET2, Ten-eleven translocation methylcytosine dioxygenase 2; TET3, Ten-eleven translocation methylcytosine dioxygenase 3; TDG, Thymine DNA Glycosylase; MBD4, Methyl-CpG-binding domain protein 4; GADD45a, Growth arrest and DNA damage-inducible proteins 45 alpha; DNMT1, DNA methyltransferase 1; DNMT3A, DNA methyltransferase 3A.
